# Analysis of risk factors and pregnancy outcomes in pregnant women with subchorionic hematoma

**DOI:** 10.1097/MD.0000000000035874

**Published:** 2023-11-24

**Authors:** Tiantian Xu, Weiwei Lun, Pengran Wang, Yuanfang He

**Affiliations:** a Department of Obstetrics, The Third Affiliated Hospital of Zhengzhou University, Zhengzhou, China.

**Keywords:** immune abnormalities, neonatal outcomes, pregnancy complications, subchorionic hematoma

## Abstract

Subchorionic hemorrhage (SCH) or hematoma is one of the abnormal ultrasonic manifestations. At present, there are few studies on the pathogenesis of SCH, and its underlying mechanism is still unclear. It may be related to abnormal placenta formation and implantation, autoimmune dysfunction, and coagulation dysfunction. As a unique complication of pregnancy, SCH has a controversial effect on pregnancy outcome. The aim of the present study was to explore the possible etiology of SCH, especially its association with autoimmune dysfunctions, as well as the pregnancy outcomes of SCH patients. This retrospective cohort study was conducted at the Third Affiliated Hospital of Zhengzhou University. Patients with a singleton pregnancy of ≤14 weeks gestation from June 2021 to June 2022 were included. Patients with SCH detected by ultrasound were selected as the study group, while patients without SCH during the same period were chosen as the control group. Immunological indicators and pregnancy outcomes were primarily compared between the 2 groups. The decrease in protein S activity and antithrombin-III levels, the increase in homocysteine levels, and the presence of autoantibodies (such as lupus anticoagulant, anticardiolipin antibody, and antinuclear antibody spectrum) were found to be risk factors for SCH. SCH in the first trimester was associated with higher rates of premature rupture of membranes (13.5% vs 3.8%) and miscarriage (14.4% vs 6.4%). However, there were no significant differences in the rates of placental abruption, fetal distress, cesarean section, neonatal birth weight, and gestational age. The incidence of miscarriage was also significantly higher in patients with subchorionic hematoma (SCH) who tested positive for autoantibodies (28.2% vs 7.6%). There were no significant differences in other clinical characteristics and pregnancy outcomes between patients with SCH who had positive autoantibodies and those who did not. The occurrence of SCH may be related to maternal immune abnormalities. SCH may increase the risk of premature rupture of membranes and abortion. However, there is no correlation between the presence or absence of SCH and neonatal outcomes.

## 1. Introduction

Subchorionic hematoma (SCH) refers to the separation and bleeding of the chorion and decidua basica, resulting in the accumulation of blood between the chorion and decidua basica, forming a hematoma. On ultrasound, SCH can be manifested as a hypoechoic or anechoic crescent shaped area between the uterine cavity and the gestational sac.^[1]^ They are thought to result from partial detachment of the chorionic membrane from the uterine wall.^[2]^ The incidence of SCH ranges from 0.46% to 39.5%.^[3]^ The significant variations in its clinical incidence are primarily attributed to factors such as the population being diagnosed, ultrasound technology, and the gestational weeks at which it is detected. Some patients with SCH in early pregnancy have no obvious symptoms, but often show symptoms such as lower abdominal pain or vaginal bleeding when combined with threatened abortion. Although SCH is relatively common, its exact etiology and clinical significance are still controversial. On the one hand, more and more studies on SCH have been conducted in recent years, and some studies have found that the etiology of SCH may be related to abnormal maternal immune factors and the formation of prethrombotic state. On the other hand, some studies have reported that the presence of SCH increases the risk of adverse pregnancy outcomes such as abortion, premature delivery and premature rupture of membranes, for example, Li Y and Alijotas J et al found that compared with normal pregnant women, the positive rate of autoantibodies such as anti-nuclear antibodies and anti-cardiolipin in SCH patients increased.^[4,5]^ Several studies have reported the effect of SCH on pregnancy outcomes, such as SCH was associated with an increased risk of spontaneous abortion, preterm birth and premature rupture of membranes (PROM).^[6,7]^ The exact etiology of SCH is still unclear. Current studies have shown that maternal immune disorders are also one of the causes of SCH. Therefore, this study mainly discusses the immune factors and pregnancy outcomes, in order to provide new ideas for the treatment of clinical SCH and reduce the occurrence of adverse pregnancy outcomes from the perspective of immunology.

## 2. Patients and methods

### 2.1. Patients

We conducted retrospective matched control study in our hospital, the study included patients with singleton pregnancies of ≤14 weeks gestation from June 2021 to June 2022. Patients with SCH detected by ultrasound were included in the study group, without SCH served as the control group. The inclusion criteria were singleton alive intrauterine pregnancy with gestational age ≤14 weeks and with or without SCH, Pregnant women aged 18–45 years. Patients were excluded gestational age >14 weeks, multiple pregnancy, known hypertensive status, uterine anomaly, cases of detected congenital anomalies, severe medical and surgical pathology. Those who lost to follow up for delivery were also excluded from the selected patients. Both groups were compared for pregnancy outcomes including miscarriage, APH including abruptio placentae & placenta previa, preterm labor, PROM and mode of delivery. In patients whose pregnancies continued and resulted in delivery, term or preterm status, birth weight, Apgar score, NICU admission and neonatal death were also compared between the 2 groups. Differences in pregnancy outcomes between autoantibody-positive and autoantibody-negative patients in the SCH group were also assessed. For those who gave birth in our hospital, we completed the follow-up work by consulting their medical records. We made telephone follow-ups to get information of SCH’s progress and their pregnancy outcomes if they took antenatal examinations and gave births in other hospitals.

### 2.2. Study design

Immunoassay results were collected from all eligible patients, Protein S activity (PS), protein C activity (PC), antinuclear antibody, anticardiolipin antibody (ACL), homocysteine (HCY), and lupus anticoagulant were detected. Antinuclear antibody was detected using indirect immunofluorescence. ACL, PS, PC, and Antiprothrombin-III (AT-III) were detected using enzyme-linked immunosorbent assay. HCY was detected using the cyclic enzymatic method. All operations were carried out in strict accordance with the instructions of the instrument, and all experimental results were provided by the experimenter.

### 2.3. Diagnostic criteria

The diagnostic criteria of subchorionic hematoma refer to “Ultrasound Atlas of Obstetrics and Gynecology.”^[8]^ Ultrasound images showed crescent-shaped or triangular areas that appeared anechoic or hypoechoic. Miscarriage is defined as the loss of a pregnancy before 28 weeks of gestation. Preterm birth is defined as a birth <37 weeks of gestation and is further categorized into early preterm birth (28–32 weeks), moderate preterm birth (32–34 weeks), and late preterm birth (34–37 weeks). Pregnancy complications, such as premature rupture of membranes, placental abruption, fetal distress, fetal growth restriction, and chorioamnionitis, were also recorded.

### 2.4. Statistical analysis

Statistical analysis of the clinical data was performed by SPSS Statistics version 26.0. Continuous data were presented as mean ± standard deviation (SD) and compared with Student *t* test. Categorical data were presented as proportion (percentage) and compared with Chi-square test or Fisher exact test where appropriate. Multivariable logistic regressions were performed with the immune abnormalities (PS, PC, AT-III, fibrinogen degradation products, and so on) as dependent variables and the SCH or non-SCH group as independent variables. Odds ratios (OR) and 95% confidence intervals (CI) were calculated in multivariable analyses. *P* values of <.05 were considered statistically significant.

## 3. Results

**Figure 1.**
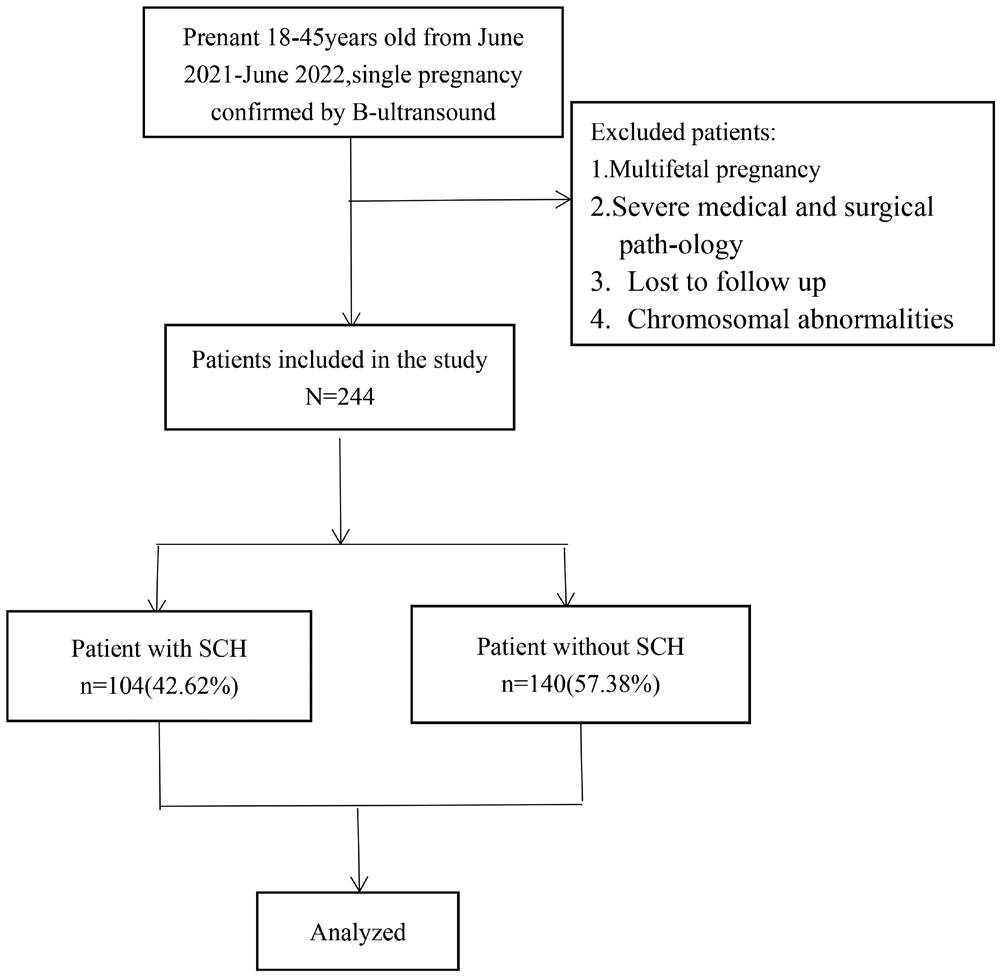
Recruitment profile of the case-control study.

A total of 112 women were detected SCH. By conducting a search for patients who underwent prenatal examinations at our hospital between June 2021 and June 2022, and by reviewing ultrasound results and screening for inclusion and exclusion criteria, we identified 104 patients who met the criteria for the SCH group, and 140 patients who met the criteria for the control group. The results are shown in Figure 1. Upon comparing the basic clinical data, no significant differences were found in age, gravidity, parity, abortion history, and assisted reproductive conception between the 2 groups (*P* > .05; Table 1).

**Table 1:** Characteristics of pregnant women with and without SCH.

Demographics	Study group (n = 104)	Control group (n = 140)	*P* value
Age (yr)	30.99 ± 3.63	30.86 ± 2.64	.741
Gestational age (wk)	8.99 ± 7.35	9.05 ± 3.06	.882
Number of pregnancies	2.07 ± 1.26	1.94 ± 0.61	.308
Order of birth	0.64 ± 0.92	0.70 ± 0.59	.568
Loss history [n (%)]	30 (28.8%)	27 (19.3%)	.081
IVF [n (%)]	14 (13.5%)	10 (7.1%)	.101

IVF = assisted reproduction.

By comparing the immune-related indexes of the 2 groups, it was found that the protein S activity and the value of antithrombin AT-III in the study group were lower than those in the normal control group. Additionally, the value of homocysteine was higher in the study group compared to the control group. These differences between the 2 groups were statistically significant (*P* < .05). The positive rates of lupus anticoagulant (10.6% vs 5.0%), ACL (13.5% vs 5.0%), and antinuclear antibody (15.4% vs 5.7%) in the SCH group were significantly higher than those in the control group (*P* < .05). However, there was no significant difference in fibrin degradation products, D-dimer, and protein C activity between the 2 groups. The incidence of vaginal bleeding was also significantly higher in the study group (25.0% vs 7.9%; Table 2)

**Table 2:** Comparison of immune indexes and vaginal bleeding between the 2 groups.

Statistical indicators	Study group (n = 104)	Control group (n = 140)	*P* value
PS	56.62 ± 25.05	70.72 ± 26.91	<.001
PC	103.43 ± 19.56	107.44 ± 15.83	.078
AT-III	93.26 ± 10.49	97.34 ± 9.84	.002
FDP	1.04 ± 0.61	0.93 ± 0.51	.255
DD	0.42 ± 0.18	0.38 ± 0.15	.139
LA [n (%)]	11 (10.6%)	7 (5.0%)	.009
ACL [n (%)]	14 (13.5%)	7 (5.0%)	.020
ANAs [n (%)]	16 (15.4%)	8 (5.7%)	.012
HCY	8.18 ± 3.66	6.43 ± 2.85	<.001
Vaginal bleeding [n (%)]	26 (25.0%)	11 (7.9%)	<.001

ACL = anticardiolipin antibody, ANAs = anti-nuclear antibody spectrum, AT-III = antiprothrombin-III, DD = D-dimer, FDP = fibrinogen degradation products, HCY = homocysteine, LA = lupus anticoagulant, PC = protein C activity, PS = protein S activity.

Binary logistic regression analysis showed that protein S activity, autoantibodies (lupus anticoagulant, ACL, and antinuclear antibody spectrum), and homocysteine were independent risk factors for SCH (Table 3).

**Table 3:** Analysis of independent risk factors for the occurrence of SCH.

Statistical indicators	*b*	*sb*	*P* value	95% CI
PS	0.023	0.007	.001	1.010–1.037
PC	0.016	0.009	.078	0.998–1.035
AT-III	0.028	0.017	.094	0.995–1.062
FDP	−0.277	0.270	.304	0.447–1.286
DD	1.090	1.185	.358	0.291–0.365
LA	1.279	0.598	.033	1.112–11.601
ACL	1.472	0.555	.008	1.469–12.927
ANAs	1.306	0.524	.013	1.323–10.307
HCY	−0.180	0.051	.000	0.756–0.923

ACL = anticardiolipin antibody, ANAs = anti-nuclear antibody spectrum, AT-III = antiprothrombin-III, DD = D-dimer, FDP = fibrinogen degradation products, HCY = homocysteine, LA = lupus anticoagulant, PC = protein C activity, PS = protein S activity.

Compared to the control group, the study group had a significantly higher rate of premature rupture of membranes (13.5% vs 3.8%) and abortion (14.4% vs 6.4%), but a significantly lower rate of term birth (73.1% vs 87.1%). There were no significant differences between the 2 groups in the rates of cesarean section (43.8% vs 33.6%), placental abruption (6.7% vs 3.1%), fetal intrauterine distress (11.2% vs 5.3%), and fetal growth restriction (9.0% vs 4.0%). There were no significant differences in birth weight, gestational age at delivery, neonatal morbidity (11.2% vs 5.4%), NICU admission (7.9% vs 3.8%), and Apgar score at 1 minute between the 2 groups (Table 4).

**Table 4:** Comparison of pregnancy complications and pregnancy outcomes between the 2 groups.

Statistical indicators	Study group (n = 104)	Control group (n = 140)	*P* value
Obstetric complications			
Premature rupture of membrane [n (%)]	12 (13.5%)	5 (3.8%)	.008
Placental abruption [n (%)]	6 (6.7%)	4 (3.1%)	.197
Fetal distress in utero [n (%)]	10 (11.2%)	7 (5.3%)	.108
Intrauterine growth restriction [n (%)]	8 (9.0%)	6 (4.6%)	.189
Chorioamnionitis [n (%)]	5 (5.6%)	3 (2.3%)	.196
Pregnancy outcomes			
Full term delivery [n (%)]	76 (73.1%)	122 (87.1%)	.005
Preterm delivery [n (%)]	13 (12.5%)	9 (6.4%)	.102
Miscarriage [n (%)]	15 (14.4%)	9 (6.4%)	.038
Mode of delivery			
Natural birth [n (%)]	50 (56.2%)	87 (66.4%)	.124
C-section [n (%)]	39 (43.8%)	44 (33.6%)	
Condition of the newborn			
Birth weight (g)	3208.42 ± 270.59	3221.83 ± 261.12	.410
Gestational age at delivery (wk)	37.91 ± 1.16	38.05 ± 1.21	.110
1 min Apgar score	9.87 ± 0.37	9.90 ± 0.32	.455
Neonatal morbidity [n (%)]	10 (11.2%)	7 (5.4%)	.112
NICU admission [n (%)]	7 (7.9%)	5 (3.8%)	.199

Table 5 comparison is shown between SCH patients with positive and negative autoantibodies. No significant differences were observed in maternal age, gestational age at diagnosis of SHC, vaginal bleeding, and assisted reproduction between the 2 groups. The full-term delivery rate of the autoantibody-positive group was lower than that of the antibody-negative group, and the abortion rate was significantly higher. Although the birth rate was lower in the antibody-positive group, there were no significant differences in birth weight, gestational age at delivery, and Apgar score at 1 minute between the 2 groups.

**Table 5:** Comparison between SCH patients with and without autoantibodies.

Statistical indicators	Autoantibody positive (n = 39)	Autoantibody negative (n = 66)	*P* value
Age (yr)	30.54 ± 3.39	31.36 ± 3.83	.269
Gestational age (wk)	59.95 ± 16.48	64.48 ± 16.32	.173
Vaginal bleeding [n (%)]	7 (17.9%)	19 (29.2%)	.198
IVF [n (%)]	6 (15.4%)	8 (12.3%)	.656
Full term delivery [n (%)]	23 (59.0%)	53 (80.3%)	.018
Preterm delivery [n (%)]	4 (10.3%)	9 (13.6%)	.611
Miscarriage [n (%)]	11 (28.2%)	5 (7.6%)	.004
Birth weight (g)	3140.74 ± 283.43	3199.66 ± 279.54	.365
Gestational age at delivery (wk)	37.62 ± 1.09	37.75 ± 1.19	.623
1 min Apgar score	9.84 ± 0.26	9.93 ± 0.41	.316

## 4. Discussion

SCH is one of the common ultrasound abnormalities observed during pregnancy. With the increasing detection rate, numerous studies have investigated the pathogenic factors and pathogenesis of this condition. However, its etiology remains unclear due to its complexity. Some studies^[9]^ have found that assisted reproductive conception is a risk factor for SCH. In this study, although the rate of assisted reproductive conception was higher in the SCH group compared to the control group, there was no statistically significant difference between the 2 groups. This lack of significance may be attributed to the small sample size of the study. Therefore, in the future, the sample size needs to be expanded to further investigate the relationship between assisted reproductive conception and SCH.

Pregnancy is a process of maternal immune tolerance to the embryo, and successful pregnancy depends on the balance of the maternal immune system and endocrine system. In recent years, research on the etiology of SCH has revealed that maternal immune abnormalities and the development of a prethrombotic state are risk factors for the occurrence of SCH. AT-III, PS, and PC are commonly used indicators of clinical coagulation function. AT-III inhibits the function of various activated coagulation factors by binding with heparin and effectively controls the imbalance between coagulation and anticoagulation.^[10]^ However, both PS and PC can inhibit coagulation factors V and VIII, thereby exerting a coagulation function and effectively preventing the continuous development of the coagulation process in the blood vessels.^[11]^ Thus, maintaining the balance of these 3 factors plays an important role in inhibiting the hypercoagulable state in the body. HCY is a sulfur-containing amino acid that does not participate in protein synthesis, and its abnormal elevation is one of the common causes of adverse pregnancy outcomes. HCY can activate coagulation factors V and VII, increase thromboxane levels, reduce the activities of coagulation factors III and IV, disrupt the balance between coagulation and anticoagulation systems, and result in a hypercoagulable state of the blood. Increased risk of thrombosis.^[12]^ This study found that the levels of PS activity and AT-III were lower in the SCH group compared to the normal control group. Additionally, the HCY level was higher in the SCH group than in the control group. Therefore, it can be speculated that the abnormal number of the 3 in the SCH group may disrupt the balance of the maternal coagulation system, causing the body’s blood to be in a hypercoagulable state. This can potentially lead to the formation of hematomas.

It has been suggested that the occurrence of SCH may be related to abnormalities in maternal autoantibodies. Antiphospholipid antibodies include anticardiolipin antibodies, lupus anticoagulant, and anti-β2 glycoprotein antibodies. ACL is a type of procoagulant substance that binds to phospholipids and competes for phospholipid receptors in placental blood vessels. This binding can cause local microthrombosis, which in turn affects the blood supply to the endometrium. Consequently, it can impact the uterus’s ability to accept the embryo, potentially leading to abortion.^[13]^ At present, the pathogenesis of adverse pregnancy outcomes caused by antinuclear antibodies is still unclear. Some studies suggest that antinuclear antibodies can lead to hyperimmunity and activate the complement pathway, which can hinder embryo development.^[14]^ Li et al^[15]^ conducted a study that included 130 normal pregnant women and 90 patients with SCH. The results revealed that the prevalence of autoantibodies in SCH patients was remarkably high, reaching 45.36%. Specifically, the positive rates of anticardiolipin antibodies and antinuclear antibodies were higher in the SCH group compared to the normal control group. At the same time. Alijotas et al^[16]^ found that when there were autoantibodies in pregnant women, especially antiphospholipid antibodies, played a certain role in the formation of intrauterine hematoma. The conclusion of the above study is similar to that of the present study, that is, the positive rate of autoantibodies in patients with SCH is higher than that in the control group, which can be used to speculate that the presence of autoantibodies may play a role in the formation of SCH.

The impact of SCH on pregnancy outcomes remains controversial. There are conflicting conclusions about the effect of SCH on pregnancy complications. This study found that the incidence of premature rupture of membranes was higher in the SCH group, while there was no significant difference in other pregnancy complications. This conclusion is similar to the results of Tuuli et al^[4]^ which found that the presence of SCH increased the incidence of premature rupture of membranes. Our study found that the miscarriage rate in SCH group was significantly higher than that in the control group, but the preterm birth rate was not significantly different, which was similar to the conclusion of some current studies. For example, a meta-analysis conducted by Li Qing et al^[17]^ found that the abortion rate in the SCH group was higher than that in the control group. However, there was no significant difference in the rates of preterm birth and cesarean section between the 2 groups. A meta-analysis study by Yan et al also concluded that the presence of SCH did not increase the incidence of preterm birth. A large retrospective cohort study by Gu et al^[18]^ found that SCH during pregnancy did not elevate the risk of negative pregnancy outcomes. A large retrospective cohort study by Gu et al^[19]^ found a higher miscarriage rate in the SCH group than in the normal group, the vaginal bleeding rate was also significantly higher. This study also find the incidence of vaginal bleeding was significantly higher in the SCH group. However, there are some different views from the conclusion of the study; a large study by Naert et al^[20]^ found that the occurrence of SCH during pregnancy did not increase the risk of adverse pregnancy outcomes. Fu et al^[6]^ also suggested that SCH does not increase the risk of adverse pregnancy outcomes and neonatal adverse conditions. Our data suggest that SCH presenting in the first trimester does not adversely affect neonatal outcomes, similar to the conclusions of several current studies.^[7,21]^ However, some studies^[22]^ have found that the presence of SCH can lead to an increased risk of small gestational age infants. At present, there is no consensus on the relationship between SCH and adverse pregnancy outcomes. The varying conclusions of each study may be attributed to differences in study design or sample size. Therefore, more prospective and large-scale experiments are needed to further clarify the relationship between SCH and adverse pregnancy outcomes in the future.

The pregnancy outcomes of patients in the SCH group, with and without autoantibodies, showed that the abortion rate was higher in patients with positive autoantibodies compared to those without autoantibodies. However, there was no significant difference in neonatal outcomes between the 2 groups. Therefore, it is suggested that the presence of maternal autoantibodies may increase the risk of miscarriage, and appropriate drug treatment should be administered to these patients. Studies have found that anticoagulants and low-dose immunosuppressive drugs can improve the prognosis of antiphospholipid syndrome or autoimmune diseases.^[23,24]^ More large-scale and prospective studies are needed to clarify the clinical significance and treatment plan of SCH. In addition, research on the etiology of SCH, particularly the relationship between SCH and immune factors, will enhance our understanding of the disease. This, in turn, will provide more treatment options for clinical practice, alleviate anxiety among pregnant women with this condition, and prevent adverse pregnancy outcomes.

In conclusion, the occurrence of SCH may be related to maternal immune abnormalities, and SCH in early pregnancy is associated with miscarriage, but there is no relationship between SCH and neonatal outcomes. Due to the limited sample size and retrospective nature of this study, more in-depth studies are needed to further explore the role of immune disorders in the pathogenesis of SCH and the impact of SCH on pregnancy outcomes.

## Author contributions

**Data curation:** Tiantian Xu.

**Conceptualization:** Weiwei Lun.

**Funding acquisition:** Weiwei Lun.

**Resources:** Yuanfang He.

**Software:** Pengran Wang.

**Visualization:** Pengran Wang.

**Writing – original draft:** Tiantian Xu.
